# Supplementary material for: Guy1, a Y-linked embryonic signal, regulates dosage compensation in Anopheles stephensi by increasing X gene expression
Source: eLife. 2019 Mar 19;8:e43570. doi: 10.7554/eLife.43570 (PMC6440743; doi:10.7554/eLife.43570)
Supplement: Supplementary file 1. [file elife-43570-supp1.docx]

**Supplementary File S1. Spearman correlation analyses of the two RNA-Seq experiments**

**Experiment A: Four biological replicates each of transgenic and wild type sibling females in line *nGuy1_2*.**

|  | **TF1** | **TF2** | **TF3** | **TF4** | **WF1** | **WF2** | **WF3** | **WF4** |
| --- | --- | --- | --- | --- | --- | --- | --- | --- |
| **TF1** | 1.00 | 0.98 | 0.98 | 0.98 | 0.98 | 0.98 | 0.99 | 0.98 |
| **TF2** | 0.98 | 1.00 | 1.00 | 1.00 | 0.99 | 0.99 | 0.99 | 0.98 |
| **TF3** | 0.98 | 1.00 | 1.00 | 1.00 | 0.99 | 0.99 | 0.99 | 0.98 |
| **TF4** | 0.98 | 1.00 | 1.00 | 1.00 | 0.99 | 0.99 | 0.98 | 0.98 |
| **WF1** | 0.98 | 0.99 | 0.99 | 0.99 | 1.00 | 1.00 | 1.00 | 0.99 |
| **WF2** | 0.98 | 0.99 | 0.99 | 0.99 | 1.00 | 1.00 | 1.00 | 0.99 |
| **WF3** | 0.99 | 0.99 | 0.99 | 0.98 | 1.00 | 1.00 | 1.00 | 0.99 |
| **WF4** | 0.98 | 0.98 | 0.98 | 0.98 | 0.99 | 0.99 | 0.99 | 1.00 |

The correlation matrix was derived from a regularized log transformed read count table, which has been normalized by size factor for each library using the R package DESeq2.

TF stands for transgenic female and WF stands for wild type female.

**Experiment B: Three biological replicates each of transgenic and wild type siblings, females and males in line *nGuy1_1*.**

|  | **WF1** | **WF2** | **WF3** | **TM1** | **TM2** | **TM3** | **TF1** | **TF2** | **TF3** | **WM1** | **WM2** | **WM3** |
| --- | --- | --- | --- | --- | --- | --- | --- | --- | --- | --- | --- | --- |
| **WF1** | 1.00 | 0.96 | 0.98 | 0.92 | 0.92 | 0.96 | 0.93 | 0.96 | 0.95 | 0.94 | 0.94 | 0.95 |
| **WF2** | 0.96 | 1.00 | 0.99 | 0.98 | 0.98 | 0.99 | 0.98 | 1.00 | 1.00 | 0.99 | 0.99 | 1.00 |
| **WF3** | 0.98 | 0.99 | 1.00 | 0.96 | 0.96 | 0.98 | 0.97 | 0.99 | 0.99 | 0.98 | 0.98 | 0.99 |
| **TM1** | 0.92 | 0.98 | 0.96 | 1.00 | 1.00 | 0.98 | 0.98 | 0.98 | 0.98 | 0.98 | 0.98 | 0.98 |
| **TM2** | 0.92 | 0.98 | 0.96 | 1.00 | 1.00 | 0.98 | 0.98 | 0.98 | 0.98 | 0.99 | 0.98 | 0.98 |
| **TM3** | 0.96 | 0.99 | 0.98 | 0.98 | 0.98 | 1.00 | 0.97 | 0.98 | 0.98 | 0.97 | 0.97 | 0.98 |
| **TF1** | 0.93 | 0.98 | 0.97 | 0.98 | 0.98 | 0.97 | 1.00 | 0.99 | 0.99 | 0.99 | 0.99 | 0.99 |
| **TF2** | 0.96 | 1.00 | 0.99 | 0.98 | 0.98 | 0.98 | 0.99 | 1.00 | 1.00 | 0.99 | 0.99 | 0.99 |
| **TF3** | 0.95 | 1.00 | 0.99 | 0.98 | 0.98 | 0.98 | 0.99 | 1.00 | 1.00 | 0.99 | 0.99 | 1.00 |
| **WM1** | 0.94 | 0.99 | 0.98 | 0.98 | 0.99 | 0.97 | 0.99 | 0.99 | 0.99 | 1.00 | 1.00 | 1.00 |
| **WM2** | 0.94 | 0.99 | 0.98 | 0.98 | 0.98 | 0.97 | 0.99 | 0.99 | 0.99 | 1.00 | 1.00 | 1.00 |
| **WM3** | 0.95 | 1.00 | 0.99 | 0.98 | 0.98 | 0.98 | 0.99 | 0.99 | 1.00 | 1.00 | 1.00 | 1.00 |

The correlation matrix was derived from a regularized log transformed read count table, which has been normalized by size factor for each library using the R package DESeq2.

TF stands for transgenic female and WF stands for wild type female.

TM stands for transgenic male and WM stands for wild type male
