# Supplementary material for: Guy1, a Y-linked embryonic signal, regulates dosage compensation in Anopheles stephensi by increasing X gene expression
Source: eLife. 2019 Mar 19;8:e43570. doi: 10.7554/eLife.43570 (PMC6440743; doi:10.7554/eLife.43570)
Supplement: Supplementary file 2. — This file shows results from analyses of pooled biological replicates. [file elife-43570-supp2.docx]

**Supplementary File S2. Analyses of median FPKMs of X-linked genes vs. autosomal genes. This file shows results from analyses of pooled biological replicates. Analyses of each individual replicate are shown in Supplementary File S3. FPKMs are derived from hisat2-stringtie.**

**Experiment A: Four biological replicates each of transgenic and wild type sibling females in line *nGuy1_2*.**

***Guy1* Transgenic Females**

| The median expression level of genes on Chromosome X and Autosomes of *Guy1* transgenic females on different FPKM cutoffs. This table of statistics does not include one of the four transgenic female replicate TF1. | | | |
| --- | --- | --- | --- |
| Cutoffs | Chromosome X | Autosomes | *p* value* |
| original | 47.4461345 | 29.257568 | 2.2e-16 |
| remove FPKM=0 | 49.942261 | 32.4558045 | 2.2e-16 |
| remove FPKM<1 | 53.595314 | 36.5692955 | 2.2e-16 |
| remove FPKM<2 | 55.406776 | 38.8460885 | 1.959e-15 |
| remove FPKM<3 | 56.116566 | 40.033707 | 3.282e-14 |
| remove FPKM<4 | 58.22279 | 41.248455 | 1.016e-14 |
| * The *p* values were calculated based on two-tailed two-sample Wilcoxon rank sum test. | | | |

***Guy1* Wild Type Sibling Females**

| The median expression level of genes on Chromosome X and Autosomes of *Guy1* wildtype females on different FPKM cutoffs. | | | |
| --- | --- | --- | --- |
| Cutoffs | Chromosome X | Autosomes | *p* value* |
| original | 29.763337 | 29.724943 | 0.2375 |
| remove FPKM=0 | 31.861872 | 32.683357 | 0.9837 |
| remove FPKM<1 | 35.366852 | 36.9405995 | 0.3149 |
| remove FPKM<2 | 37.29336 | 39.035391 | 0.1653 |
| remove FPKM<3 | 39.416973 | 40.280338 | 0.2525 |
| remove FPKM<4 | 41.583462 | 41.414005 | 0.5561 |
| * The *p* values were calculated based on two-tailed two-sample Wilcoxon rank sum test. | | | |

**Experiment B: Three biological replicates each of transgenic and wild type siblings, females and males in line *nGuy1_1*.**

***Guy1* Transgenic Females**

| The median expression level genes on Chromosome X and Autosomes of *Guy1* transgenic females on different FPKM cutoffs. | | | |
| --- | --- | --- | --- |
| Cutoffs | Chromosome X | Autosomes | *p* value* |
| original | 38.010929 | 24.848562 | 2.2e-16 |
| remove FPKM=0 | 41.3626195 | 28.459894 | 1.359e-13 |
| remove FPKM<1 | 44.6884535 | 31.630104 | 5.402e-12 |
| remove FPKM<2 | 47.331646 | 33.001965 | 4.007e-12 |
| remove FPKM<3 | 49.219803 | 34.306984 | 4.219e-13 |
| remove FPKM<4 | 50.150002 | 35.282219 | 1.993e-12 |
| * The *p* values were calculated based on two-tailed two-sample Wilcoxon rank sum test. | | | |

***Guy1* Wild Type Sibling Females**

| The median expression level genes on Chromosome X and Autosomes of *Guy1* wildtype females on different FPKM cutoffs . | | | |
| --- | --- | --- | --- |
| Cutoffs | Chromosome X | Autosomes | *p* value* |
| original | 22.824542 | 23.183908 | 0.6775 |
| remove FPKM=0 | 25.959938 | 28.044546 | 0.2182 |
| remove FPKM<1 | 28.6443815 | 31.631308 | 0.09681 |
| remove FPKM<2 | 31.4810655 | 34.25642 | 0.0785 |
| remove FPKM<3 | 33.5783025 | 36.294147 | 0.3037 |
| remove FPKM<4 | 36.199131 | 38.250675 | 0.2876 |
| * The *p* values were calculated based on two-tailed two-sample Wilcoxon rank sum test. | | | |

***Guy1* Transgenic Males**

| The median expression level genes on Chromosome X and Autosomes of *Guy1* transgenic males on different FPKM cutoffs . | | | |
| --- | --- | --- | --- |
| Cutoffs | Chromosome X | Autosomes | *p* value* |
| original | 28.274393 | 28.236641 | 0.1575 |
| remove FPKM=0 | 30.26759 | 31.532209 | 0.7795 |
| remove FPKM<1 | 32.418987 | 34.204247 | 0.3678 |
| remove FPKM<2 | 33.866436 | 35.647856 | 0.3667 |
| remove FPKM<3 | 34.952587 | 36.7715015 | 0.2724 |
| remove FPKM<4 | 35.414803 | 37.78112 | 0.187 |
| * The *p* values were calculated based on two-tailed two-sample Wilcoxon rank sum test. | | | |

***Guy1* Wild Type Sibling Males**

| The median expression level genes on Chromosome X and Autosomes of *Guy1* wildtype males on different FPKM cutoffs . | | | |
| --- | --- | --- | --- |
| Cutoffs | Chromosome X | Autosomes | *p* value* |
| original | 26.735091 | 25.095482 | 0.02227 |
| remove FPKM=0 | 30.560925 | 29.009514 | 0.3899 |
| remove FPKM<1 | 32.781242 | 32.1466215 | 0.9048 |
| remove FPKM<2 | 34.586437 | 33.62463 | 0.7995 |
| remove FPKM<3 | 35.6759265 | 34.719982 | 0.7849 |
| remove FPKM<4 | 36.4169615 | 35.769196 | 0.7547 |
| * The *p* values were calculated based on two-tailed two-sample Wilcoxon rank sum test. | | | |
