# Supplementary material for: Guy1, a Y-linked embryonic signal, regulates dosage compensation in Anopheles stephensi by increasing X gene expression
Source: eLife. 2019 Mar 19;8:e43570. doi: 10.7554/eLife.43570 (PMC6440743; doi:10.7554/eLife.43570)
Supplement: Supplementary file 3. [file elife-43570-supp3.docx]

**Supplementary File S3. Analyses of median FPKMs of X-linked genes vs. autosomal genes of *Guy1* transgenic and sibling wild type males and females: Individual replicates.**

**Experiment A**

**(Includes four biological replicates each of transgenic and wild type sibling females from line *nGuy1_2)***

| The median expression level genes on Chromosome X and Autosomes of individual replicates on different FPKM cutoffs. | | | | |
| --- | --- | --- | --- | --- |
| Sample | **Cutoffs** | **Chromosome X** | **Autosomes** | ***p* value*** |
| TF1 | original | 32.73911 | 25.36624 | 0.0000032 |
|  | remove FPKM=0 | 34.23133 | 28.27414 | 0.0000929 |
|  | remove FPKM<1 | 38.12126 | 33.37074 | 0.0012021 |
|  | remove FPKM<2 | 39.12130 | 35.62863 | 0.0080437 |
|  | remove FPKM<3 | 40.19464 | 37.32130 | 0.0241118 |
|  | remove FPKM<4 | 41.54838 | 38.98843 | 0.0157060 |
| TF2 | original | 48.09706 | 29.34398 | 0.0000000 |
|  | remove FPKM=0 | 50.02313 | 31.72866 | 0.0000000 |
|  | remove FPKM<1 | 54.33247 | 36.23512 | 0.0000000 |
|  | remove FPKM<2 | 56.06585 | 38.18527 | 0.0000000 |
|  | remove FPKM<3 | 57.27402 | 39.56045 | 0.0000000 |
|  | remove FPKM<4 | 59.61916 | 40.57501 | 0.0000000 |
| TF3 | original | 47.25824 | 29.48545 | 0.0000000 |
|  | remove FPKM=0 | 48.84125 | 31.81949 | 0.0000000 |
|  | remove FPKM<1 | 53.00118 | 36.08971 | 0.0000000 |
|  | remove FPKM<2 | 54.85021 | 38.09955 | 0.0000000 |
|  | remove FPKM<3 | 56.71317 | 39.62318 | 0.0000000 |
|  | remove FPKM<4 | 57.48009 | 40.86897 | 0.0000000 |
| TF4 | original | 48.27311 | 28.42878 | 0.0000000 |
|  | remove FPKM=0 | 49.66551 | 30.69105 | 0.0000000 |
|  | remove FPKM<1 | 54.14014 | 34.74229 | 0.0000000 |
|  | remove FPKM<2 | 55.87047 | 36.80736 | 0.0000000 |
|  | remove FPKM<3 | 57.11069 | 38.24527 | 0.0000000 |
|  | remove FPKM<4 | 58.94167 | 39.39848 | 0.0000000 |
| WF1 | original | 33.21823 | 31.93921 | 0.1096829 |
|  | remove FPKM=0 | 34.36693 | 33.94951 | 0.5060686 |
|  | remove FPKM<1 | 38.07142 | 38.49541 | 0.7098231 |
|  | remove FPKM<2 | 39.07339 | 40.57882 | 0.2595766 |
|  | remove FPKM<3 | 39.80928 | 41.93657 | 0.0979172 |
|  | remove FPKM<4 | 40.58271 | 43.10473 | 0.1173107 |
| WF2 | original | 31.32496 | 30.03590 | 0.1396375 |
|  | remove FPKM=0 | 32.90151 | 32.70109 | 0.5870737 |
|  | remove FPKM<1 | 36.15963 | 36.88966 | 0.6335401 |
|  | remove FPKM<2 | 37.54853 | 39.04405 | 0.2235345 |
|  | remove FPKM<3 | 37.92733 | 40.53806 | 0.0821774 |
|  | remove FPKM<4 | 40.11088 | 41.75763 | 0.0969756 |
| WF3 | original | 28.38357 | 28.93329 | 0.3400655 |
|  | remove FPKM=0 | 30.21288 | 31.34326 | 0.9962674 |
|  | remove FPKM<1 | 33.74510 | 35.75736 | 0.2684612 |
|  | remove FPKM<2 | 34.91657 | 38.01898 | 0.0591080 |
|  | remove FPKM<3 | 35.83335 | 39.63836 | 0.0152332 |
|  | remove FPKM<4 | 36.98058 | 40.95549 | 0.0181708 |
| WF4 | original | 27.80146 | 26.81764 | 0.6772408 |
|  | remove FPKM=0 | 28.50499 | 28.97513 | 0.5663081 |
|  | remove FPKM<1 | 30.98920 | 32.89602 | 0.0860183 |
|  | remove FPKM<2 | 31.76021 | 34.73761 | 0.0122936 |
|  | remove FPKM<3 | 32.50901 | 36.01238 | 0.0022122 |
|  | remove FPKM<4 | 34.41250 | 37.33669 | 0.0026995 |
| * The *p* values were calculated based on two-tailed two-sample Wilcoxon rank sum test. | | | | |

TF stands for transgenic female and WF stands for wild type female.

**Experiment B**

**(Includes three biological replicates each of transgenic and wild type siblings, females and males from line *nGuy1_1)***

| The median expression level genes on Chromosome X and Autosomes of individual replicates on different FPKM cutoffs. | | | | |
| --- | --- | --- | --- | --- |
| Sample | **Cutoffs** | **Chromosome X** | **Autosomes** | ***p* value*** |
| TF1 | original | 44.11868 | 24.81068 | 0.0000000 |
|  | remove FPKM=0 | 47.96569 | 28.01255 | 0.0000000 |
|  | remove FPKM<1 | 51.27411 | 30.93878 | 0.0000000 |
|  | remove FPKM<2 | 52.57228 | 32.35107 | 0.0000000 |
|  | remove FPKM<3 | 54.69685 | 33.40814 | 0.0000000 |
|  | remove FPKM<4 | 55.78713 | 34.45700 | 0.0000000 |
| TF2 | original | 34.83820 | 24.45009 | 0.0000000 |
|  | remove FPKM=0 | 36.68801 | 27.90732 | 0.0000001 |
|  | remove FPKM<1 | 40.00901 | 31.29029 | 0.0000014 |
|  | remove FPKM<2 | 42.46854 | 33.28070 | 0.0000046 |
|  | remove FPKM<3 | 44.36842 | 34.44827 | 0.0000012 |
|  | remove FPKM<4 | 45.51521 | 35.56911 | 0.0000037 |
| TF3 | original | 35.29282 | 24.67383 | 0.0000000 |
|  | remove FPKM=0 | 37.62919 | 27.93297 | 0.0000000 |
|  | remove FPKM<1 | 42.61275 | 30.99172 | 0.0000000 |
|  | remove FPKM<2 | 44.23310 | 32.57421 | 0.0000001 |
|  | remove FPKM<3 | 45.79959 | 33.86423 | 0.0000000 |
|  | remove FPKM<4 | 46.41808 | 35.03189 | 0.0000000 |
| WF1 | original | 15.27519 | 17.47472 | 0.4757489 |
|  | remove FPKM=0 | 17.48922 | 21.75904 | 0.0154931 |
|  | remove FPKM<1 | 19.93687 | 26.03838 | 0.0010549 |
|  | remove FPKM<2 | 21.24736 | 28.03097 | 0.0002239 |
|  | remove FPKM<3 | 23.63765 | 30.00622 | 0.0003438 |
|  | remove FPKM<4 | 24.38594 | 31.68233 | 0.0001159 |
| WF2 | original | 25.93906 | 25.10549 | 0.1757534 |
|  | remove FPKM=0 | 28.29876 | 29.09910 | 0.7609926 |
|  | remove FPKM<1 | 31.38840 | 32.45869 | 0.2451113 |
|  | remove FPKM<2 | 33.14708 | 34.35158 | 0.0987620 |
|  | remove FPKM<3 | 35.23736 | 35.68372 | 0.1131287 |
|  | remove FPKM<4 | 35.83109 | 37.01462 | 0.0492647 |
| WF3 | original | 22.00316 | 23.15549 | 0.8994348 |
|  | remove FPKM=0 | 24.02845 | 26.83179 | 0.1112036 |
|  | remove FPKM<1 | 27.88774 | 30.58598 | 0.0118488 |
|  | remove FPKM<2 | 28.34537 | 32.86205 | 0.0026012 |
|  | remove FPKM<3 | 29.83758 | 34.25192 | 0.0032826 |
|  | remove FPKM<4 | 30.39321 | 35.71824 | 0.0008946 |
| TM1 | original | 28.45515 | 27.36612 | 0.0315941 |
|  | remove FPKM=0 | 30.60138 | 30.30378 | 0.3942956 |
|  | remove FPKM<1 | 33.01025 | 32.73546 | 0.9996444 |
|  | remove FPKM<2 | 33.95889 | 34.06868 | 0.8009359 |
|  | remove FPKM<3 | 35.08437 | 34.99059 | 0.9908024 |
|  | remove FPKM<4 | 35.25238 | 35.70688 | 0.8120714 |
| TM2 | original | 29.80197 | 28.04263 | 0.0325376 |
|  | remove FPKM=0 | 31.28594 | 31.18841 | 0.3877313 |
|  | remove FPKM<1 | 33.31816 | 33.57231 | 0.9508447 |
|  | remove FPKM<2 | 34.75718 | 34.97070 | 0.8783554 |
|  | remove FPKM<3 | 35.75629 | 35.85164 | 0.9182568 |
|  | remove FPKM<4 | 36.03081 | 36.63975 | 0.9019879 |
| TM3 | original | 26.14090 | 28.02450 | 0.9043343 |
|  | remove FPKM=0 | 28.87129 | 32.05259 | 0.1044891 |
|  | remove FPKM<1 | 31.09175 | 35.61514 | 0.0078921 |
|  | remove FPKM<2 | 32.50085 | 37.34024 | 0.0029296 |
|  | remove FPKM<3 | 33.82129 | 38.51816 | 0.0056113 |
|  | remove FPKM<4 | 34.50024 | 39.52242 | 0.0025702 |
| WM1 | original | 27.63828 | 25.22931 | 0.0098123 |
|  | remove FPKM=0 | 30.21914 | 28.43178 | 0.1690694 |
|  | remove FPKM<1 | 32.91796 | 31.01218 | 0.5348675 |
|  | remove FPKM<2 | 33.86776 | 32.47843 | 0.6765864 |
|  | remove FPKM<3 | 35.52263 | 33.42565 | 0.4913381 |
|  | remove FPKM<4 | 36.05396 | 34.12339 | 0.6284832 |
| WM2 | original | 26.31373 | 24.52034 | 0.0181919 |
|  | remove FPKM=0 | 29.23738 | 27.57237 | 0.2630817 |
|  | remove FPKM<1 | 31.83280 | 30.31282 | 0.7449153 |
|  | remove FPKM<2 | 32.74433 | 31.72449 | 0.9013076 |
|  | remove FPKM<3 | 33.23448 | 32.53960 | 0.6903028 |
|  | remove FPKM<4 | 34.04633 | 33.36122 | 0.8357315 |
| WM3 | original | 26.57025 | 25.13356 | 0.0666469 |
|  | remove FPKM=0 | 28.32810 | 28.83634 | 0.5880701 |
|  | remove FPKM<1 | 31.01017 | 31.48132 | 0.7721826 |
|  | remove FPKM<2 | 33.04681 | 32.97658 | 0.5964656 |
|  | remove FPKM<3 | 34.28299 | 34.03079 | 0.7903757 |
|  | remove FPKM<4 | 34.97951 | 34.87732 | 0.6388842 |
| * The *p* values were calculated based on two-tailed two-sample Wilcoxon rank sum test. | | | | |

TF stands for transgenic female and WF stands for wild type female.

TM stands for transgenic male and WM stands for wild type male.
