# Supplementary material for: Guy1, a Y-linked embryonic signal, regulates dosage compensation in Anopheles stephensi by increasing X gene expression
Source: eLife. 2019 Mar 19;8:e43570. doi: 10.7554/eLife.43570 (PMC6440743; doi:10.7554/eLife.43570)
Supplement: Supplementary file 5. [file elife-43570-supp5.docx]

**Supplementary File S5. Significantly up-regulated and down-regulated genes on chromosome X and autosomes between *Guy1* transgenic and wild type sibling males and females.**

**Experiment A**

**(Includes four biological replicates each of transgenic and wild type sibling females)**

| The number of significant* up- and down-regulated genes on Chromosome X | | | | |
| --- | --- | --- | --- | --- |
| and autosomes comparing *Guy1* transgenic to wild type females. | | | | |
|  | up-regulated | down-regulated | non-regulated | *p* value (Chi-square test) |
| Chr X | 580 | 27 | 389 | 2.20e-16 |
| Autosomes | 1214 | 1658 | 7479 |  |
| * BH adjusted *p* value < 0.1 | | |  |  |

The ratio of up-regulated to down-regulated genes is approximately 29 fold [(580/27)/(1214/1658)] greater for the X chromosome compared to autosomes.

**Experiment B**

**(Includes three biological replicates each of transgenic and wild type siblings, females and males)**

| The number of significant* up- and down-regulated genes on chromosome X | | | | |
| --- | --- | --- | --- | --- |
| and autosomes comparing *Guy1* transgenic to wild type females. | | | | |
|  | up-regulated | down-regulated | non-regulated | *p* value (Chi-square test) |
| Chr X | 475 | 12 | 509 | 2.20e-16 |
| Autosomes | 701 | 1206 | 8444 |  |
| * BH adjusted *p* value < 0.1 | | |  |  |

The ratio of up-regulated to down-regulated genes is approximately 68 fold [(475/12)/(701/1206)] greater for the X chromosome compared to autosomes.

| The number of significant* up- and down-regulated genes on chromosome X | | | | |
| --- | --- | --- | --- | --- |
| and autosomes comparing *Guy1* transgenic to wild type males. | | | | |
|  | up-regulated | down-regulated | non-regulated | *p* value (Chi-square test) |
| Chr X | 91 | 130 | 775 | 2.17e-02 |
| Autosomes | 1240 | 1228 | 7883 |  |
| * BH adjusted *p* value < 0.1 | | |  |  |

The ratio of up-regulated to down-regulated genes is 0.69 fold [(91/130)/(1240/1228)] when comparing the X chromosome to autosomes.
